# *In vivo* analysis of highly conserved Nef activities in HIV-1 replication and pathogenesis

**DOI:** 10.1186/1742-4690-10-125

**Published:** 2013-10-30

**Authors:** Richard L Watkins, Wei Zou, Paul W Denton, John F Krisko, John L Foster, J Victor Garcia

**Affiliations:** 1 Division of Infectious Diseases, Center for AIDS Research, 2042 Genetic Medicine, University of North Carolina, Campus Box 7042, Chapel Hill, NC 27599-7042, USA

**Keywords:** HIV-1, Nef, Replication, Pathogenesis, BLT humanized mice, Mutation

## Abstract

**Background:**

The HIV-1 accessory protein, Nef, is decisive for progression to AIDS. *In vitro* characterization of the protein has described many Nef activities of unknown *in vivo* significance including CD4 downregulation and a number of activities that depend on Nef interacting with host SH3 domain proteins. Here, we use the BLT humanized mouse model of HIV-1 infection to assess their impact on viral replication and pathogenesis and the selection pressure to restore these activities using enforced *in vivo* evolution.

**Results:**

We followed the evolution of HIV-1_LAI_ (LAI) with a frame-shifted *nef* (LAINef*fs*) during infection of BLT mice. LAINef*fs* was rapidly replaced in blood by virus with short deletions in *nef* that restored the open reading frame (LAINef*fs*∆-1 and LAINef*fs*∆-13). Subsequently, LAINef*fs*∆-1 was often replaced by wild type LAI. Unexpectedly, LAINef*fs*∆-1 and LAINef*fs*∆-13 Nefs were specifically defective for CD4 downregulation activity. Viruses with these mutant *nef*s were used to infect BLT mice. LAINef*fs*∆-1 and LAINef*fs*∆-13 exhibited three-fold reduced viral replication (compared to LAI) and a 50% reduction of systemic CD4^+^ T cells (>90% for LAI) demonstrating the importance of CD4 downregulation. These results also demonstrate that functions other than CD4 downregulation enhanced viral replication and pathogenesis of LAINef*fs*∆-1 and LAINef*fs*∆-13 compared to LAINef*fs*. To gain insight into the nature of these activities, we constructed the double mutant P72A/P75A. Multiple Nef activities can be negated by mutating the SH3 domain binding site (P72Q73V74P75L76R77) to P72A/P75A and this mutation does not affect CD4 downregulation. Virus with *nef* mutated to P72A/P75A closely resembled the wild-type virus *in vivo* as viral replication and pathogenesis was not significantly altered. Unlike LAINef*fs* described above, the P72A/P75A mutation had a very weak tendency to revert to wild type sequence.

**Conclusions:**

The *in vivo* phenotype of Nef is significantly dependent on CD4 downregulation but minimally on the numerous Nef activities that require an intact SH3 domain binding motif. These results suggest that CD4 downregulation plus one or more unknown Nef activities contribute to enhanced viral replication and pathogenesis and are suitable targets for anti-HIV therapy. Enforced evolution studies in BLT mice will greatly facilitate identification of these critical activities.

## Background

Patients infected with *nef*-defective HIV-1, have strongly attenuated viral replication and pathogenesis [1-4]. *In vitro* studies have defined numerous Nef activities but how this 206 amino acid protein has such a major effect on the outcome of HIV-1 infection in patients is unknown [5-9]. One view of Nef’s overall impact on HIV-1 infection is that there is a cumulative effect of multiple activities to achieve high viral loads resulting in the development of AIDS [10,11]. In support of this view, a number of Nef activities have been found to be conserved in monkey, ape and human immunodeficiency viruses [12-17]. A difficulty with this interpretation is that there are so many Nef activities that the effect of any given activity on replication and pathogenesis would be small. Alternatively, one or a few Nef functionalities may be the major contributors to viral replication and pathogenesis. In this regard CD4 downregulation, a highly conserved Nef function, is of particular interest. *Ex vivo* studies with activated peripheral blood T cells and cultures of tonsil tissue support a dominant role for CD4 downregulation in establishing high rates of viral replication [18-20]. Another factor that may be critical is the SH3 domain binding site in Nef’s polyproline helix [21-23]. This ten amino acid segment (PVRPQVPLRP) is the most highly conserved stretch of amino acids in the protein [24]. Evidence exists for SH3 domain binding site involvement with enhanced viral replication [21,23,25], cytotoxic effects [26-30], activation of Hck [31] and antagonism of host immune responses [32-36]. Nef structure/function studies have documented that the CD4 downregulation activity and the SH3 domain protein dependent activities are genetically distinct [21,37,38].

To gain greater understanding of the roles of Nef’s diverse activities during HIV-1 replication we have employed the BLT humanized mouse model. This model has stable reconstitution of a full spectrum of human immune cells and has been used to investigate a number of different aspects of HIV-1 infection [39-44]. With regard to Nef, we have previously compared the replicative properties of HIV-1_LAI_ (LAI) and LAI with two large deletions in *nef* coding sequence (LAINef*dd*) in BLT humanzed mice [44]. LAI exhibited high levels of viral replication and near total depletion of CD4^+^ T cells in blood and tissues, as well as, depletion of CD4^+^ CD8^+^ thymocytes from the human thymic organoid. LAINef*dd* had significantly reduced viral replication and dramatically reduced capacity for inducing CD4^+^ T cell and CD4^+^ CD8^+^ thymocyte loss [44]. However, one important aspect of HIV-1 infection of BLT humanized mice that has not yet been investigated is the ability of *nef* to evolve during HIV-1 infection. In patients, HIV-1 *nef* extensively mutates resulting in tremendous sequence diversity but it has not been possible to clearly relate these changes to Nef activities or the pathogenic potential of the virus [24,45-49]. Here, we investigate three critical features of Nef’s role during HIV-1 infection: 1) the ability of the virus to mutate *nef* sequences to gain enhanced replicative fitness, 2) the role of CD4 downregulation in viral replication and pathogenesis and 3) the importance of Nef’s interactions with host SH3 domain proteins in replication and pathogenesis. We find that Nef induced CD4 downregulation is highly significant for active viral replication and pathogenesis. In addition, there are unidentified function(s) that contribute to viral replication and/or CD4^+^ T cell depletion and are necessary for Nef’s full pathogenic potential. Importantly, this latter function or functions does not depend on interactions with host cell SH3 domain proteins.

## Results

**Figure 1:**
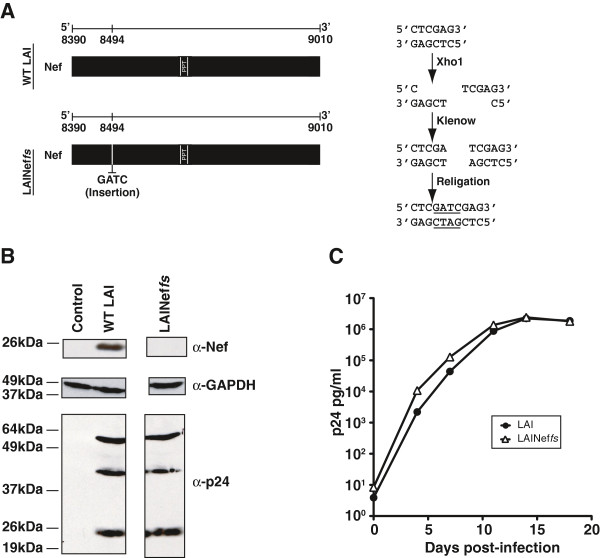
**A frame shift in *nef *ablates expression of Nef but does not affect viral replication. (A)***Upper Panel, Left*, Schematic representation of wild type LAI (WT LAI) is presented. Nucleotides 8390 to 9010 in NCBI accession number, K02013, represent the *nef* coding sequence. *Lower Panel*, *Left*, A schematic of frame shifted *nef* (LAINef*fs*) is presented. The insertion of GATC following nucleotide 8494 is indicated. PPT, polypurine tract. *Upper Panel*, *Right,* Flow chart describing the generation of the GATC insertion is presented. **(B)** The frame shift in *nef* eliminates Nef expression but does not alter the expression of Gag. *nef* (α-Nef) and *gag* (α-p24) encoded proteins were detected by Western blot analysis of 293T producer cell lysates. GAPDH (α-GAPDH) is a protein loading control. **(C)** A3.01 cells were infected with LAI or LAINef*fs* at a multiplicity of infection of 0.05 and viral production was followed for 20 days with ELISA for p24^*gag*^.

With the exceptions of *vif* and *pol*, the ability of HIV-1 to correct defective genes *in vivo* and regain function has not been investigated [50,51]. Determining the selection pressure for Nef functions is a key component for characterizing the overall importance of Nef and the phenotypic contribution of its individual activities. In addition, it provides an opportunity to use enforced selection to discern what structural/functional motifs of Nef are important *in vivo*. To address the mutational response of the virus to a defective *nef*, we generated an inactivating mutation by filling in the 5′ four-base overhang generated by the XhoI restriction endonuclease at codon 35 with Klenow (Figure 1A and Additional file 1). This insertion resides 5′ of the polypurine tract and does not alter the synthesis of *gag* encoded proteins (Figure 1B) or alter the *in vitro* replication properties of the virus (Figure 1C).

**Figure 2:**
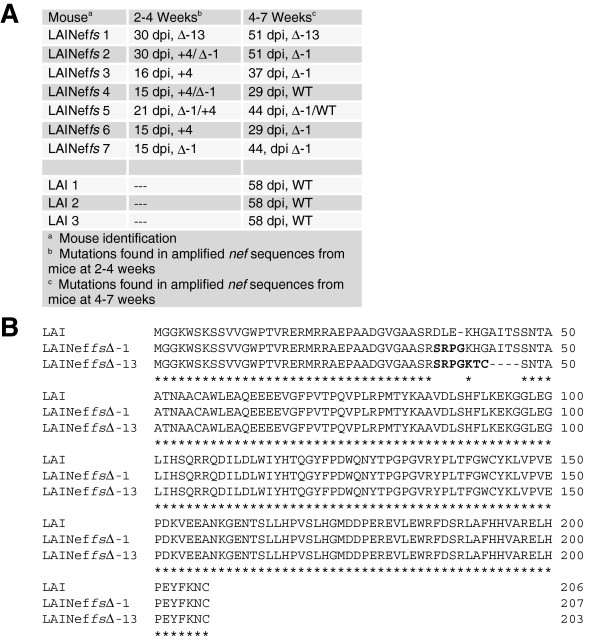
**LAINef*fs *mutates to have one of three *nefs *with an open reading frame. (A)** At several time points during the eight week infection, viral RNA was reverse transcribed from plasma. After amplification by PCR, *nef* was sequenced. By four weeks, six of seven mice exhibited *nef* sequence with a one base deletion just downstream of the four base insertion in a run of five adenines. This one base deletion (∆-1) restored the *nef* reading frame. In the remaining mouse, a 13 base deletion occurred downstream of the four base insertion (∆-13) which also restored the *nef* open reading frame. Three of the seven mice had a mixture of two different sequences in plasma which are indicated by the “majority sequence/the minority sequence.” By seven weeks, there were two mice with reversions to wild type sequence (WT) by the removal of four inserted bases from CTCGATCGAG to yield CTCGAG. +4, *nef* with the original four base insertion intact. **(B)** The conceptual translations of the LAINef*fs*∆-1 and LAINef*fs*∆-13 mutated *nef*s are aligned with wild type LAI Nef amino acid sequence. Bold indicates missense amino acids encoded by LAINef*fs*∆-1 and LAINef*fs*∆-13.

LAI and the *nef* frame-shifted LAI (LAINef*fs*) were injected intravenously (iv, 360,000 TCIU) into BLT mice. Three LAI infected mice were monitored over eight weeks for virus in the blood. These mice had peak viral loads of 12.2 ± 4.7 × 10^6^ copies of viral RNA. No changes were observed in *nef* sequence after eight weeks of the infection (Figure 2A). The LAINef*fs* inoculated mice were also monitored longitudinally for the presence of virus in plasma. LAINef*fs* infected mice exhibited active viral replication with peak viral loads of 2.26 ± 0.72 × 10^6^ copies of viral RNA per ml of blood (n = 7). We sequenced *nef* from viral RNA in blood from two to eight weeks post-infection and found that the frame-shifted LAINef*fs* (designated “+4”) was initially replaced by one of two *nef* sequences with restored open reading frames (Figure 2A). One mouse (LAINef*fs* 1) had a thirteen base deletion downstream of the original four base insertion (∆-13). In the other six cases (LAINef*fs* 2–7), the *nef* coding sequence also retained the four base insertion but lost one base in a run of five adenines just downstream of the original insertion site (∆-1). The original LAINef*fs* (+4) was not detectable in blood by five weeks post infection in any of the seven mice. In two mice, and by a slower process, wild type *nef* appeared by 4–7 weeks (LAINef*fs* 4, 5). After eight weeks, four mice (LAINef*fs* 3, 4, 5 and 6) were predominantly infected with a virus containing wild type *nef* (not shown). In summary, in the blood of all of the mice inoculated with LAINef*fs* the original defective *nef* was replaced by a *nef* mutant that restored the open reading frame. Sequences determined at eight weeks yielded four mice with exact removal of the four base insertion (WT), two mice with ∆-1 and one mouse with ∆-13. These results support the conclusion that a strong positive selection exists for a functioning *nef*.

The amino acid sequences of the restored *nef*s are reported in Figure 2B. The changes in Nef sequence resulting from the one base deletion (LAINef*fs*∆-1) and the thirteen base deletion (LAINef*fs*-13) were the replacement of three amino acids (DLE, 36–38) in wild type LAINef with four missense amino acids (SRPG) and the replacement of ten wild type amino acids (DLEKHGAITS, 36–45) with seven missense amino acids (SRPGKTC), respectively (Figure 2B). The sequencing data suggested that virus with *fs*∆-1 and *fs*∆-13 *nef*s had a strong replicative advantage over the *nef*-defective virus. However, the replacement of LAINef*fs*∆-1 with wild type (WT) virus in four mice further suggests a replicative advantage for wild type *nef* over revertant *nef*s. Based on these *in vivo* findings, we were interested in characterizing the *in vitro* activities of the ∆-1 and ∆-13 mutant Nefs.

### *In vitro* functional analysis of *nef* mutants that evolved *in vivo*

**Figure 3:**
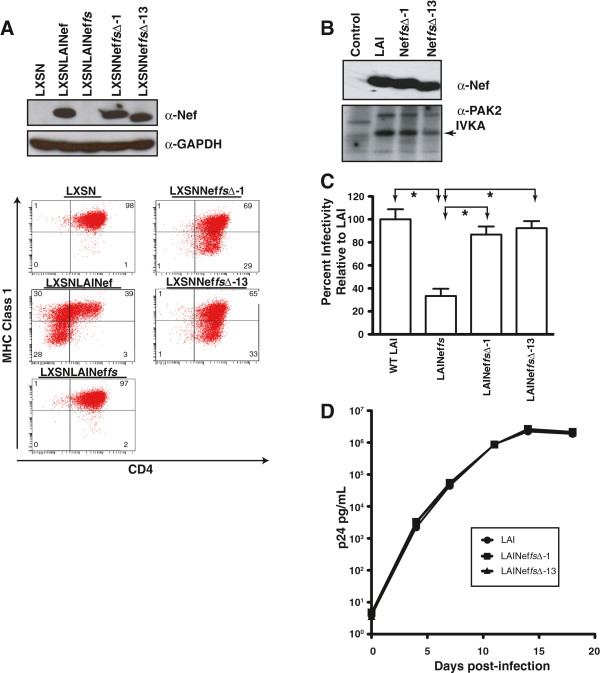
**LAINef*fs*Δ - 1 and LAINef*fs*Δ - 13 encode Nefs that are specifically defective for downregulating surface expression of CD4. (A)** Nefs encoded by LAINef*fs*∆-1, LAINef*fs*∆-13 and LAI were expressed in CEM cells following transduction with retroviral vectors (LXSN). *Upper Panel*, A Western blot demonstrates LAI Nef*fs*∆-1 and LAI Nef*fs*∆-13 were expressed at comparable levels as wild type (α-Nef). LXSN and LXSNNef*fs* served as negative controls. GAPDH is a protein loading control (α-GAPDH). *Lower Panel*, CEM cells expressing LAI Nef, LAI Nef*fs*, LAI Nef*fs*∆-1 and LAI Nef*fs*∆-13 were analyzed by flow cytometry for cell surface CD4 and MHC Class I (MHCI) expression. LXSNLAINef was the positive control. LXSN and LXSNLAINef*fs* were negative controls. Percentage of cells in each quadrant out of total cells is indicated. **(B)** Nefs encoded by LAI, LAINef*fs*∆-1 and LAINef*fs*∆-13 were expressed in transfected 293T cells. Control, 293T cells transfected with empty vector. *Upper Panel*, Lysates from transfected cells were analyzed by Western blot (α-Nef). *Lower Panel*, Total p21 activated protein kinase-2 (PAK2) in lysates of transfected cells lysates were immunoprecipitated with anti-PAK2 antiserum (α-PAK2) and analyzed by the *in vitro* kinase assay (IVKA). Control cells had no activated PAK2. Arrow, autophosphorylated PAK2. **(C)** pLAI, pLAINef*fs*, pLAINef*fs*∆-1 and pLAINef*fs*∆-13 proviral clones were transfected into 293T cells and virus harvested from the media. LAI, LAINef*fs*, LAINef*fs*∆-1 and LAINef*fs*∆-13 were titered using HeLa-MAGI indicator cells [82] and p24^*gag*^ contents were quantified by ELISA. Infectivities (blue cells per ng p24^*gag*^) from six determinations of each virus were normalized relative to LAI (100%). Significant comparisons are indicated by lines and arrows above respective bars (*, p < 0.05). **(D)** A3.01 cells were infected with LAI, LAINef*fs*∆-1 and LAINef*fs*∆-13 at multiplicity of infection of 0.05 and viral production followed for 20 days with ELISA for p24^*gag*^.

To assess *in vitro* phenotypes of the Nefs expressed by LAINef*fs*∆-1 and LAINef*fs*∆-13, we transferred the coding sequences into the retroviral expression plasmid, pLXSN, and produced retroviral vectors [52]. CEM T cells expressing wild type and mutant Nefs were assayed for level of expression, CD4 downregulation and MHC Class I (MHCI) downregulation activities (Figure 3A). The mutant forms of Nef were expressed at the same level as wild type Nef (Figure 3A, *Upper Panel*, α-Nef). Flow cytometric analysis of cell surface CD4 and MHCI expression of CEM T cells that were transduced to express LAI Nef yielded the well-known patterns for the downregulation of these proteins (Figure 3A, *Lower Panel*, [21,52,53]). Both LAI Nef*fs*∆-1 and LAI Nef*fs*∆-13 proteins were fully active for MHCI downregulation but devoid of CD4 downregulation activity. To assess the effect of the two mutations on Nef’s interaction with p21 activated protein kinase (PAK2), we expressed the Nef*fs*∆-1 and Nef*fs*∆-13 proteins from pcDNA3.1 in transfected 293T cells. We determined the capacity of these Nefs to activate PAK2 using an *in vitro* kinase assay (IVKA, [21,54,55]). Again, both mutant proteins were expressed at the same level as wild type LAI Nef (Figure 3B, α-Nef) and both proteins activated PAK2 although at a reduced level for LAI Nef*fs*∆-13 (Figure 3B, α-PAK2 IVKA). We also generated the proviral clones, pLAINef*fs*∆-1 and pLAINef*fs*∆-13, to characterize the enhancement of infectivity function of these Nefs. Virus was produced from transfected 293T cells and assayed with HeLa MAGI indicator cells. In this single infection assay, a reduction in the number of infected cells per ng of virion p24^*gag*^ is observed for LAINef*fs* relative to LAI (Figure 3C). LAINef*fs*∆-1 and LAINef*fs*∆-13 both exhibited higher infectivities than LAINef*fs* in this assay but were not significantly different from LAI (Figure 3C). Finally, in Figure 3D, the capacities of LAINef*fs*∆-1 and LAINef*fs*∆-13 to replicate in A3.01 cells were observed to be the same as LAI (Figure 3D). On the basis of this data, we concluded that LAINef*fs*∆-1 and LAINef*fs*∆-13 exhibited a specific loss of the CD4 downregulation activity and were potentially useful to investigate the impact of CD4 downregulation by Nef on HIV-1 infection in BLT mice. However, the question remained whether these *nef*s could revert to wild type sequence *in vivo* as four of seven mice infected with LAINef*fs* had predominantly the wild type *nef* sequence in blood after eight weeks (Figure 2A). It should be noted that reversion of the · ∆-1 mutation to wild type would require two steps, a four-base deletion and a one base insertion of adenosine. We judged this two-step process to be unlikely to occur within the time frame of the experiments. Accordingly, the wild type *nef*s found in four of the seven mice by week eight may have been directly generated from the frame-shifted *nef* in LAINef*fs* by the exact removal of the four-base insertion (Additional file 1).

### Infection of BLT humanized mice with *in vivo* generated *nef* mutations

**Figure 4:**
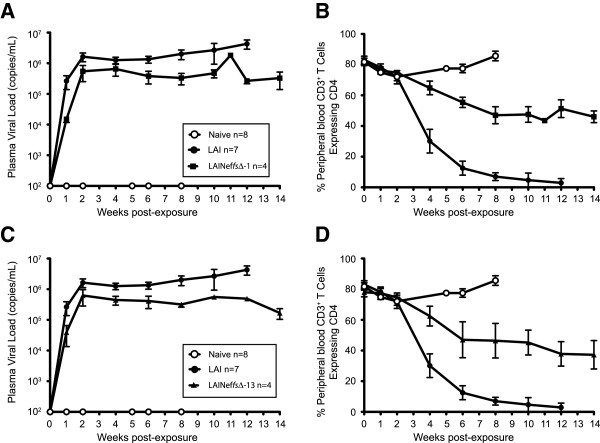
**Viral load analysis and peripheral blood CD4**^**+ **^**T cell depletion in mice infected with LAI. (A)** Viral loads (copies of LAI RNA per milliliter of plasma) of BLT humanized mice exposed to 90,000 TCIU of LAI or LAINef*fs*∆-1 were plotted. Uninfected mice (Naïve) served as negative controls (open circle, n = 8); LAI (filled circle, n = 7); and LAINef*fs*∆-1 (filled square, n = 4). **(B)** Plot of percent peripheral blood CD3^+^ T cells expressing CD4. Naïve mice, LAI and LAINef*fs*∆-1 as in **(A)**. **(C)** Viral loads were plotted following inoculation of 90,000 TCIU of LAI or LAINef*fs*∆-13. Naïve mice as negative controls (open circle, n = 8); LAI (filled circle, n = 7); and LAINef*fs*∆-13 (filled triangles, n = 4). **(D)** Plot of percent peripheral blood CD3^+^ T cells expressing CD4. Naïve mice, LAI and LAINef*fs*∆-13 as in **(C)**.

LAI, LAINef*fs*∆-1 and LAINef*fs*∆-13 were intravenously injected (90,000 TCIU) into BLT mice. In Figure 4, the positive control, wild type LAI, and the negative control, uninfected mice (Naïve), are compared to LAINef*fs*∆-1 and LAINef*fs*∆-13 infected mice. In Figure 4A and C, LAI inoculation was followed with rapid appearance of viral RNA in blood and replication to high levels (peak viral loads, 3.03 ± 0.54 × 10^6^ copies viral RNA per ml of plasma, n = 7). The time course for the infections with LAINef*fs*∆-1 and LAINef*fs*∆-13 revealed a reduction in viral replication compared to LAI (Figure 4A and C). The average peak viral load (in millions of RNA copies per ml of peripheral blood) for LAINef*fs*∆-1 was 1.19 ± 0.26 (n = 4) which was significantly different from LAI (Figure 4A, 3.03 ± 0.54 (n = 7); p = 0.0242). Similarly, in Figure 4C, the average peak viral load for LAINef*fs*∆-13 was lower than LAI (0.93 ± 0.23 (n = 4); p = 0.0061). Thus, there appears to be about a threefold reduction in peak viral load relative to wild type for LAINef*fs*∆-1 and LAINef*fs*∆-13.

During infection with LAI, CD4^+^ T cell levels in blood were dramatically reduced (Figure 4B and D) while CD4^+^ T cells in the blood of uninfected mice were maintained at approximately 80% of total blood T cells (Figure 4B and D). For LAI, the average time to reduce CD4^+^ T cells to 50% of total blood T cells was 21.6 ± 2.4 days post infection (dpi, n = 7). For mice inoculated with LAINef*fs*∆-1 or LAINef*fs*∆-13, an intermediate loss of CD4^+^ T cells was evident (Figure 4B and D). The time for CD4^+^ T cells in blood to decline to 50% of total T cells was determined and compared to LAI (Figure 4B and D). As noted, LAI gave 21.6 ± 2.4 dpi (n = 7) which was significantly shorter than LAINef*fs*∆-1 at 65.1 ± 13.4 dpi (n = 4, p = 0.0106) and LAINef*fs*∆-13 at 52.5 ± 13.5 dpi (n = 4, p = 0.0294). LAINef*fs*∆-1 and LAINef*fs*∆-13 infected mice were not statistically different from each other. Together, the results from Figure 4 document an intermediate *in vivo* Nef phenotype for LAINef*fs*∆-1 and LAINef*fs*∆-13.

We have previously reported the phenotypes of LAI and LAI with a totally inactivated *nef* (LAINef*dd*, [44]). The observation that LAI expressing a Nef specifically defective for CD4 downregulation has an intermediate phenotype not expected based on previous reports [18,19,56]. In support of this conclusion, we also observed that a partial loss of CD4^+^ T cells from blood is established by six weeks. At this time point, the percent of CD4^+^ T cells in LAINef*fs*∆-1 and LAINef*fs*∆-13 infected mice were significantly lower than in Naïve mice but significantly higher than in LAI-infected mice (Figure 4B and D). For LAINef*fs*∆-1 inoculated mice, the percent CD4^+^ T cells of total T cells present in blood was 55.4 ± 3.3 (n = 4) compared to 77.5 ± 2.8 (n = 4) for Naïve (Figure 4B) with p = 0.0286. For LAINef*fs*∆-13 inoculated mice, the percentages were 47.0 ± 11.7 (n = 4) versus 77.5 ± 2.8 (n = 4) with p = 0.0286. Also at six weeks, LAINef*fs*∆-1 and LAINef*fs*∆-13 infected mice had higher percentages of CD4^+^ T cells than LAI infected mice (Figure 4B and D). Percent of CD4^+^ T cells for LAI was 12.5 ± 4.5 (n = 6), versus 55.4 ± 3.3 (n = 4, p = 0.0095) for LAINef*fs*∆-1. Percent of CD4^+^ T cells for LAI versus LAINef*fs*∆-13 was 12.5 ± 4.5 (n = 6) versus 47.0 ± 11.7 (n = 4, p = 0.0190).

At eight weeks, CD4^+^ T cells in blood of LAI infected mice are nearly depleted while Naïve mice maintained CD4^+^ T cells at approximately 80% of total CD4^+^ T cells (Figure 4B and D, [44,57]). It was of interest to allow the LAINef*fs*∆-1 and LAINef*fs*∆-13 infections to continue past eight weeks to determine if these viruses would slowly deplete CD4^+^ T cells from blood. By 14 weeks, substantial levels of CD4^+^ T cells were still evident in blood for both viruses which emphasizes the persistence of the partial Nef phenotype in the absence of CD4 downregulation (Figure 4B and D).

### Systemic loss of CD4^+^ T cells in BLT humanized mice infected with LAINef*fs*∆-1 and LAINef*fs*∆-13

**Figure 5:**
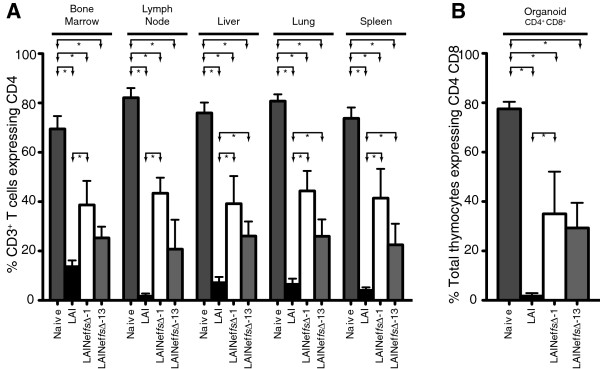
**Analysis of CD4**^**+ **^**T cells from tissues in mice exposed to LAI, LAINef*fs*Δ - 1 or LAINef*fs*Δ - 13. (A)** Percent CD4^+^ T cells of total T cells in five organs from un-exposed BLT mice (Naive, n = 8) were compared to groups of BLT mice exposed to one of three viruses: LAI (n = 6), LAINef*fs* ∆-1 (n = 4), or LAINef*fs* ∆-13 (n = 4). Statistical comparisons reaching significance are indicated by lines and arrows above respective bars (*, p < 0.05). **(B)** The same groups as in **(A)** were compared for CD4^+^CD8^+^ double positive thymocytes relative to total thymocytes.

We previously observed that systemic loss of human CD4^+^ T cells from organs closely paralleled loss of human CD4^+^ T cells from blood during infection with wild type (LAI) and *nef*-defective (LAINef*dd*) virus [44,57]. For LAINef*fs*∆-1 and LAINef*fs*∆-13 infected mice, we also determined that the loss of CD4^+^ T cells in peripheral blood is matched by the loss of these cells from bone marrow, lymph node, liver, lung and spleen (Figure 5A). Statistical analysis of Naïve versus LAI, LAINef*fs*∆-1 and LAINef*fs*∆-13 infected mice demonstrated significant losses in the percent of CD4^+^ T cells in the five tissues (fifteen comparisons to Naive, all gave p < 0.05). Also, the fraction of total T cells that were CD4^+^ was consistently higher in LAINef*fs*∆-1 infected mice compared to LAI (all five comparisons, p < 0.05). In the case of LAINef*fs*∆-13 versus LAI, three of five organs had statistically higher levels of CD4^+^ T cells in LAINef*fs*∆-13 infected mice, with the higher levels of CD4^+^ T cells not reaching statistical significance for bone marrow and lymph node. The comparisons between LAINef*fs*∆-1 and LAINef*fs*∆-13 infected mice were not significantly different in any tissue. Therefore, the partial reduction of CD4^+^ T cells in blood seen with LAINef*fs*∆-1 and LAINef*fs*∆-13 infection is systemic.

We previously reported a devastating impact of LAI infection on CD4^+^ CD8^+^ thymocytes. However, LAI lacking a functional *nef* failed to reduce double positive thymocytes [44]. In Figure 5B, drastic depletion of CD4^+^ CD8^+^ thymocytes was confirmed following inoculation with LAI. Intermediate losses were observed with LAINef*fs*∆-1 and LAINef*fs*∆-13 (Naive, 76.3 ± 3.0%; LAI, 1.7 ± 1.2%; LAINef*fs*∆-1, 35.0 ± 17.1%; LAINef*fs*∆-13, 29.3 ± 10.2%). On the basis of the above results, we conclude that the partial losses of LAINef*fs*∆-1 and LAINef*fs*∆-13 found for CD4^+^ T cells appeared to extend to CD4^+^ CD8^+^ thymocytes as well.

The mechanistic interpretation of the intermediate phenotype of the LAINef*fs*∆-1 and LAINef*fs*∆-13 viruses depends on the status of the sequence of *nef.* We sequenced *nef* in plasma virion RNA of LAINef*fs*∆-1 and LAINef*fs*∆-13 and found no reversions over the course of infection. Specifically, for LAINef*fs*∆-1, the four base insertion and the ∆-1 deletion remained intact. For LAINef*fs*∆-13, the four base insertion and the thirteen base deletion remained intact. There were no second site mutations present in *nef* either (not shown). The absence of wild type *nef* sequence from LAINef*fs*∆-1 and LAINef*fs*∆-13 infected BLT mice implies the stability of the phenotypic properties of these two *nef*s during infection. This failure of *nef*s from LAINef*fs*∆-1 and LAINef*fs*∆-13 to revert to wild type supports the hypothesis that the appearance of wild type *nef* sequence found in four of seven mice (Figure 2) infected with LAINef*fs* was the result of an exact four base deletion and not a two-step removal of the four base insertion plus a one base addition (Additional file 1). Therefore, our investigations of LAINef*fs*∆-1 and LAINef*fs*∆-13 demonstrate that LAIs stably lacking Nef’s CD4 downregulation activity have the *in vivo* phenotype of a reduced capacity for viral replication, for CD4^+^ T cell depletion and for CD4^+^ CD8^+^ thymocyte depletion relative to LAI [44].

### LAI, LAINef*fs*∆-1 and LAINef*fs*∆-13 and systemic T cell activation

One explanation for the intermediate infection phenotypes of LAINef*fs*∆-1 and LAINef*fs*∆-13 would be an inability of these mutated HIV-1 to induce systemic T cell activation [58,59]. It has been previously reported that naïve BLT mice have approximately 2% of CD8^+^ T cells that are CD38^+^ HLA-DR^+^ double positive in blood. Infection with LAI or LAINef*dd* elevates this fraction to approximately 8% [42,44]. We observed similar effects of LAINef*fs*∆-1 and LAINef*fs*∆-13 infection on T cell activation. At six weeks post infection, LAINef*fs*∆-1 and LAINef*fs*∆-13 were determined to have 8.2 ± 3.5% (n = 4) and 6.1 ± 2.3% (n = 4) CD38^+^ HLA-DR^+^ double positive CD8^+^ T cells in blood, respectively. Thus, LAINef*fs*∆-1 and LAINef*fs*∆-13 exhibit the same enhancements of peripheral blood T cell activation as LAI and LAINef*dd*.

### The role of SH3 domain dependent activities on LAI infection of BLT mice

**Figure 6:**
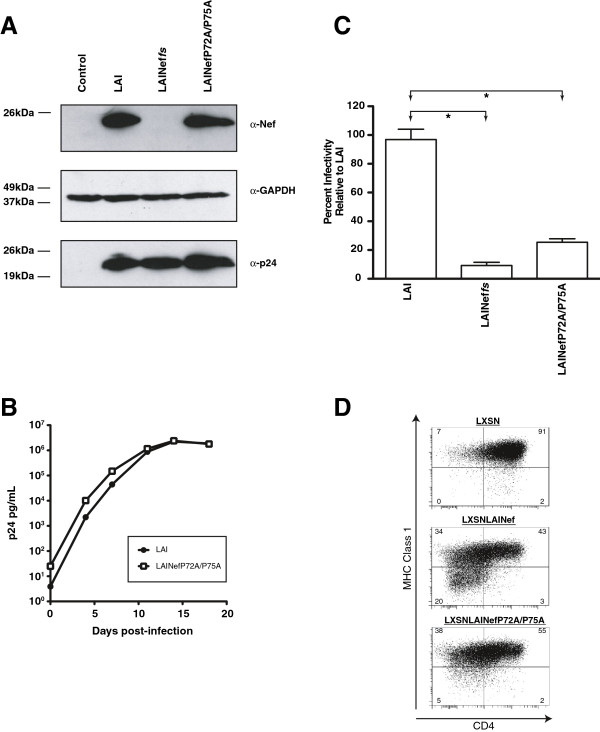
**LAINefP72A/P75A replicates in A3.01 T cells and is functional for CD4 downregulation.** pLAI, pLAINef*fs* and pLAINefP72A/P75A proviral clones were transfected into 293T cells and virus harvested from the media. **(A)** Nef (α-Nef) and p24^gag^ (α-p24) proteins were detected by Western blot analysis of 293T producer cell lysates. Control is non-transfected 293T cells. GAPDH (α-GAPDH) is a protein loading control. **(B)** A3.01 cells were infected with LAI and LAINefP72A/P75A at multiplicity of infection of 0.05 and viral production followed for 20 days with ELISA for p24^*gag*^. **(C)** LAI, LAINef*fs* and pLAINefP72A/P75A were titered using HeLa-MAGI indicator cells [82] and p24^*gag*^ quantitated by ELISA. Infectivities were normalized to LAI (100%). **(D)** Nefs encoded by LAINefP72A/P75A and LAI were expressed in CEM cells following transduction with retroviral vectors (LXSN). CEM cells expressing LAI Nef and LAI NefP72A/P75A were analyzed by flow cytometry for cell surface CD4 and MHC Class I expression. LXSN is the negative control. Percentage of cells in each quadrant out of total cells indicated.

A large number of diverse activities of Nef have been shown to be dependent on the highly conserved SH3 domain binding site. We considered the possibility that these activities may account for the observed selective advantage of the LAINef*fs*∆-1 and LAINef*fs*∆-13 over LAINef*fs* despite the absence the CD4 downregulation activity. SH3 domain-binding dependent activities are blocked by mutating two key prolines in Nef’s polyproline helix (P72A/P75A, [7,22]). To investigate the role of the P72A/P75A mutant Nef *in vivo* we generated isogenic, replication competent LAINefP72A/P75A. In 293T cells, LAINefP72A/P75A expressed similar levels of Nef and p24^*gag*^ compared to LAI (Figure 6A) and actively replicated in A3.01 T cells (Figure 6B). We assayed the enhancement of virion infectivity for LAI and LAINefP72A/P75A and observed the expected loss of this activity for the SH3 domain binding site mutant (Figure 6C, [21,25,37]). Also, we expressed the mutated *nef* from LAINefP72A/P75A with the retroviral vector, LXSN, in CEM T cells and found it to be functional for CD4 downregulation but consistent with previous reports largely defective for MHCI downregulation (Figure 6D [32,33,60]). On the basis of these results, we concluded that infecting BLT mice with LAINefP72A/P75A would distinguish between the phenotypic impacts of SH3 domain binding protein dependent activities and CD4 downregulation.

**Figure 7:**
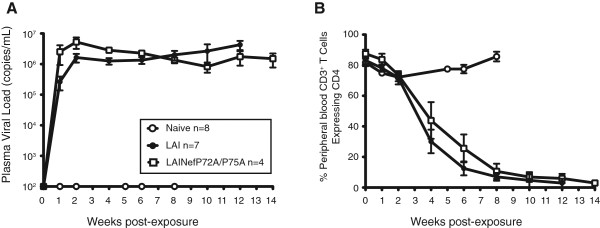
**Viral load analysis and peripheral blood CD4**^**+ **^**T cell depletion in mice infected with LAINefP72A/P75A. (A)** Viral loads of BLT mice were plotted for BLT humanized mice that were exposed to 90,000 TCIU of LAI (n = 7) and LAINefP72A/P75A (n = 4). Uninfected mice (Naïve) served as negative controls. **(B)** The percent of CD4^+^ T cells out of total T cells in peripheral blood are plotted for mice in **(A)**.

BLT mice were infected with 90,000 TCIU of LAINefP72A/P75A mutant virus (Figure 7). Under these experimental conditions, a 1.9-fold higher peak viral load was observed for LAINefP72A/P75A versus LAI (Figure 7A). This difference was not statistically different (5.83 ± 1.84 (n = 4) versus 3.03 ± 0.54 (n = 7); p = 0.1091). In addition, the P72A/P75A Nef mutant and the wild type virus showed a similar time course for reduction of peripheral blood CD4^+^ T cells to 50% of total T cells in blood with LAINefP72A/P75A at 29.5 ± 4.1 dpi (n = 4) versus LAI at 21.6 ± 2.4 dpi (n = 7); p = 0.1554 (Figure 7B). These results indicate that a functional SH3 domain binding site in Nef is not required *in vivo* for either high levels of virus replication or for CD4^+^ T cell depletion.

### Systemic depeltion of CD4^+^ T cells and thymocytes by LAINefP72A/P75A

**Figure 8:**
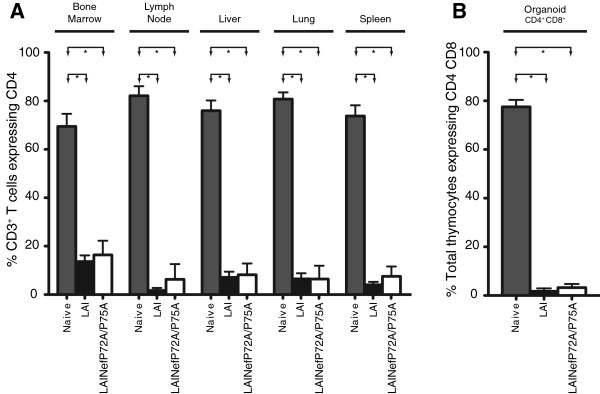
**Analysis of CD4**^**+ **^**T cells from tissues from mice exposed to LAI or LAINefP72A/P75A. (A)** Percent of CD4^+^ T cells out of total T cells from bone marrow, lymph node, liver, lung and spleen from unexposed BLT mice (Naive, n = 8) were compared to groups of BLT mice exposed to LAI (n = 7) or LAINefP72A/P75A (n = 4). Statistical comparisons reaching significance are indicated by lines and arrows above respective bars (*, p < 0.05). **(B)** The same analysis as in **(A)** is presented for CD4^+^CD8^+^ double positive thymocytes relative to total thymocytes. Statistical comparisons reaching significance are indicated by lines and arrows above respective bars (*, p < 0.05).

In transgenic mice, it has been reported that expression of HIV-1 Nef from a CD4 promoter is cytotoxic to CD4^+^ T cells in multiple organs [61]. In addition, this cytotoxic effect is lost when the polyproline helix is mutated [27]. Therefore, we determined the impact LAINefP72A/P75A infection in BLT mice on CD4^+^ T cells in bone marrow, lymph node, liver, lung and spleen (Figure 8A). LAI and LAINefP72A/P75A effectively depleted CD4^+^ T cells. All differences in levels of CD4^+^ T cells between Naïve mice and either LAI or LAINefP72A/P75A mice are statistically significant. In contrast, comparisons between the levels of residual CD4^+^ T cells in mice infected with LAI versus LAINefP72A/P75A were not significantly different (Figure 8A). CD4^+^ CD8^+^ thymocytes in the human thymic organoid were also analyzed. We found that these cells were efficiently depleted by LAINefP72A/P75A (Figure 8B). We, therefore, conclude that contrary to expectations the mutation, P72A/P75A has little to no effect on the systemic depletion of CD4^+^ T cells or CD4^+^ CD8^+^ thymocytes *in vivo*.

### *In vivo* selection pressure to correct the P72A/P75A mutation is weak

**Figure 9:**
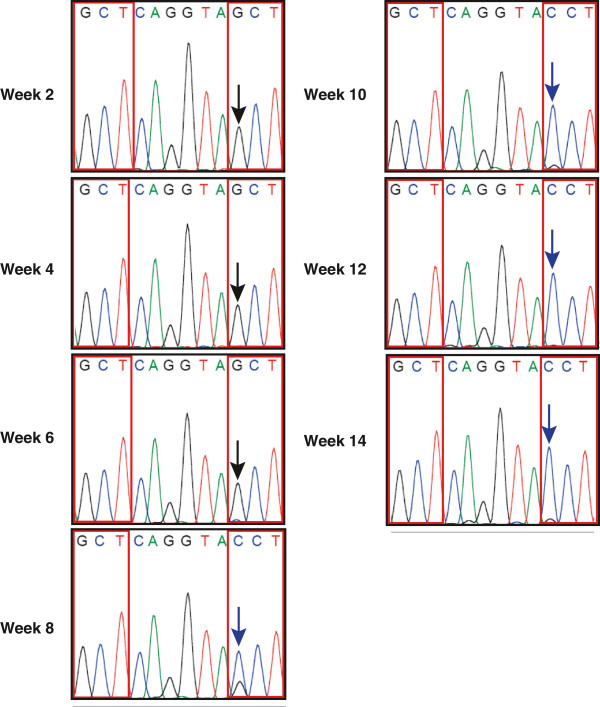
**Delayed partial reversion of P72A/P75A.***nef* sequences were obtained from viral RNA in plasma of four LAINefP72A/P75A infected mice from Figure 7. Only one of four mice had a change in *nef* sequence which is shown in the panels here. This was the reversion of the P75A mutation back to proline. The delayed and limited appearance of this mutation may be the result of the low probability of a transversion (G to C) coupled to a small enhancement of viral fitness. The twelve nucleotides encoding the SH3 domain binding motif core (P72Q73V74P75) are presented. The wild type proline codons that were mutated to alanine are P72A (left panel headed by GCT) and P75A (right panel headed by GCT). The replacement of guanine with cytosine that restores the P75 codon is seen in the right panels of weeks 8, 10, 12 and 14 headed with CCT instead of GCT. Arrow, guanine mutated to cytodine. A, green; C, blue; G, black: T, red. The four codons presented are GCT, alanine; CCT, proline; CAG, glutamine; GTA, valine.

Our conclusion that an intact SH3 domain binding site is not a major factor in determining the level of HIV-1 replication suggests that there is little or no selection pressure for reversion of the alanines to prolines. LAINefP72A/P75A virion RNA from plasma of the LAINefP72A/P75A infected mice from Figures 7 and 8 was processed for sequencing. No nucleotide changes in *nef* were noted through six weeks for the entire *nef* sequence from all four mice. Also, no changes were seen to fourteen weeks for three of four mice (not shown). At week eight, however, *nef* sequence from one of the four mice infected with the P72A/P75A mutant virus had a clear shift from guanine to mostly cytosine at the first base of the codon for position 75 (Figure 9). This transversion converted the mutant alanine codon (GCT) to the wild type proline codon (CCT). Even though the CD4^+^ T cells in LAINefP72A/P75A infected mice were nearly depleted, we continued monitoring the infection past eight weeks to determine if further mutations would occur during LAINefP72A/P75A infection. Interestingly, for the mouse presented in Figure 9, the virus with an alanine codon at 72 and proline codon at 75 completely replaced the input virus but failed to revert the alanine codon at position 72 (Figure 9, Weeks 10–14). There were no other changes in the *nef* sequence from this mouse (not shown). Since no reversion to original SH3 domain binding site (P72/P75) occurred within the eight week time frame, the high levels of viral replication and peripheral blood CD4^+^ T cell depletion could not be explained by appearance of wild type virus. Thus, our results support a model where CD4 downregulation plus one or a few additional activities- not dependent on the SH3 domain binding site- largely account for Nef’s impact on viral replication and/or pathogenesis. Loss of the capacity for SH3 domain binding has little effect on viral replication and pathogenesis and exhibits at best a small reduction in viral fitness.

## Discussion

Previously, we established that there are large phenotypic differences between infection of BLT mice with wild type LAI and the *nef*-defective LAINef*dd in vivo*[44]. LAI replicates to high viral loads concomitantly with aggressive and systematic depletion of CD4^+^ T cells and CD4^+^ CD8^+^ thymocytes. LAINef*dd* exhibits 6–7 fold lower peak viral loads and has little to no capacity to deplete CD4^+^ T cells or thymocytes [44]. These two large effects of Nef make it feasible to characterize the importance of Nef’s individual activities in BLT mice [5]. Here, we have demonstrated a third important property of *nef* in the BLT mouse model- the ability to evolve and restore functionality. Viruses expressing Nef proteins have a decisive replicative advantage over the frame-shifted LAINef*fs* and replace the *nef*-defective virus within a few weeks. Hence, in seven mice, the input LAINef*fs* was lost after four weeks with either LAINef*fs*∆-1 (six mice) or LAINef*fs*∆-13 (one mouse) being the sole virus in peripheral blood. By eight weeks, four of seven mice further evolved to be predominantly wild type virus.

The strong *in vivo* selection of LAINef*fs*∆-1 and LAINef*fs*∆-13 over LAINef*fs* led us to characterize these *in vivo* selected mutant proteins *in vitro*. We discovered them to be stable but with a total loss of CD4 downregulation activity. Three other *in vitro* Nef activities, MHCI downregulation, PAK2 activation and enhancement of virion infectivity, remained intact. When BLT mice are infected with LAINef*fs*∆-1 and LAINef*fs*∆-13, we observed an approximate 3-fold reduction in peak viral load and a partial loss of CD4^+^ T cells and CD4^+^ CD8^+^ thymocytes relative to that observed for LAI. These observations suggest that the *in vivo* selection of the two viruses with mutant *nef*s relative to LAINef*fs* relied on activities beyond CD4 downregulation. Conversely, the partial reduction of Nef effectiveness observed for LAINef*fs*∆-1 and LAINef*fs*∆-13 demonstrates a significant role for CD4 downregulation.

Our data provided evidence that there is selective pressure for restoration of Nef activities other than CD4 downregulation. The identity of these activities is unknown. We considered likely candidates to be one or more of the SH3 domain binding site dependent activities. These activities include enhancement of virion infectivity [21,25,37], PAK2 activation [21-23], upregulation of FasL and PD1 [28,29], activation of Hck [31], downregulation of MHCI [32-34] and Lck-dependent activation Ras-Erk signaling to promote the production of the T lymphocyte survival factor IL-2 [62,63]. We mutated prolines 72 and 75 to alanine to prevent interactions between Nef and host cell SH3 domain proteins [21,22]. This mutation did not exhibit a negative effect on Nef function in BLT mice. One explanation for this counter intuitive observation is that high levels of replication and rapid reduction in CD4^+^ T cell and CD4^+^ CD8^+^ thymocytes depend on only a few Nef activities.

Future studies with BLT mice will investigate Nef activities that are potentially responsible for the CD4 downregulation-independent aspects of Nef function *in vivo*. Possible activities include elevated secretion of exosomes, blocking the anti-viral effect of autophagy and inhibition of ASK1 [64-69]. Conversely, these studies may lead to the important result that known Nef activities may not account for a substantial portion of its impact on HIV-1 infection *in vivo*[70]. In this regard, our mutational strategy of introducing palindromic insertions into Nef coding sequence can be extended to scan the protein for regions of special significance for viral replication and pathogenesis. The HIV-1/BLT mice infection model described here is a feasible experimental platform for resolving these questions.

## Conclusion

CD4 downregulation activity accounts for approximately half of Nef’s capacity to enhance viral replication and deplete CD4^+^ T cells and CD4^+^ CD8^+^ thymocytes. This result is consistent with the high degree of conservation of the CD4 downregulation activity. Identities of the Nef activities that account for the remainder of Nef’s effects are unknown. We found these latter activities which are present in LAINef*fs*∆-1 and LAINef*fs*∆-13 provide the virus a strong selective advantage over LAINef*fs* that is fully defective for Nef expression. In addition, wild type virus, expressing a fully active Nef, out-competes virus expressing Nef specifically defective for CD4 downregulation. We tested the Nef activities dependent on the SH3 domain binding site because the corresponding amino acid sequence in the protein is highly conserved. However, the virus with *nef* mutated for SH3 domain binding was essentially wild type in its ability to enhance viral replication and deplete CD4^+^ T cells and CD4^+^ CD8^+^ thymocytes. Selective pressure for the mutant *nef* to revert to wild type was low. It is critical to determine which Nef activities or activities that do not depend on SH3 domain protein binding yet have major impacts on viral replication and pathogenesis.

## Methods

### Preparation of BLT humanized mice

BLT humanized mice were prepared as previously described [40-42,44,57,71-77]. Briefly, thymus/liver implanted or NOD/SCID IL-2γ^-/-^ mice (The Jackson Laboratories, Bar Harbor, ME) were transplanted with autologous human CD34^+^ cells isolated from fetal liver (Advanced Bioscience Resources, Alameda, CA). Human reconstitution in the peripheral blood of these mice was monitored periodically by flow cytometry (FACSCanto; BD Biosciences). Mice were maintained either at the Animal Resources Center, UT Southwestern Medical Center at Dallas (UTSWMC) or at the Division of Laboratory Animal Medicine, University of North Carolina at Chapel Hill (UNC-CH) in accordance with protocols approved by the UTSWMC or UNC-CH Institutional Animal Care and Use Committees.

To ensure genetic diversity, fifteen different tissue donors were used to generate five groups of mice used for the experiments presented in this manuscript. The overall level of engraftment for all the mice used in this manuscript was 60.9% ± 3.2% (n = 27). None of the groups (Naïve, LAI, LAINef*fs*∆-1, LAINef*fs*∆-13 and LAINefP72A/P75A) had significantly different engraftment levels compared to any of the other groups (p ≥ 0.1535). All groups had at least two different human genetic backgrounds included in the evaluation of infection. LAINef*fs*∆-1, LAINef*fs*∆-13 and LAINefP72A/P75A infected groups each shared a common donor with the LAI infected group.

### Cell lines and culture conditions

HeLa Magi and TZM-bl cells were maintained in Dulbecco’s modified Eagle’s medium (DMEM; Cellgro, Herndon, VA) supplemented with 10% fetal bovine serum (FBS; Cellgro), 100 IU/ml of penicillin, 100 μg/ml streptomycin, and 2 mM glutamine (Cellgro) in 10% CO_2_ at 37°C. Similarly, 293T cells were cultured under the same conditions as TZM-bl and HeLa Magi cells but in 5% CO_2_. The human CEM T cell line was cultured in RPMI 1640 medium supplemented with 10% fetal bovine serum (Hyclone), 50 IU of penicillin per ml, 50 μg streptomycin per ml, 2 mM L-glutamine and 1 mM sodium pyruvate in 10% CO_2_ at 37°C.

### Proviral clones

The proviral clone, pLAI (accession # K02013), was described by Peden et al. [78]. pLAINef*fs* was constructed to be defective for *nef* by cutting with XhoI, filling in with Klenow and re-ligating. This leaves *nef* sequence intact but introduces a four-base frame-shift after *nef* codon 35 (Additional file 1). The one base deletion (8501) and thirteen base deletion (8511–8523) found in *nef* sequences from LAINef*fs* infected mice were inserted into pLAINef *fs* by site directed mutagenesis to produce pLAINef*fs*∆-1 and pLAINef*fs*∆-13, respectively.

### Virus production, exposure of BLT mice to HIV-1_LAI_ and HIV-1_LAI_ with mutated *nef*s, tissue harvesting and flow cytometric analyses

Stocks of LAI, LAINef*fs*, LAINef*fs*∆-1, LAINef*fs*∆-13 and LAINefP72A/P75A were prepared and titered as we previously described [54,79]. Briefly, proviral clones were transfected into 293T cells. Viral supernatant was collected 48 hours after transfection and diluted in Dulbecco’s modified Eagle’s medium (DMEM) supplemented with 10% fetal bovine serum, 100 IU penicillin/ml, 100 μg/ml streptomycin, and 2 mM glutamine. TZM-bl cells were infected in 12-well tissue culture plates with 0.4 ml of virus at multiple dilutions in medium for two hours. Then, 1.0 ml of supplemented DMEM was added and the plates incubated overnight. Virus containing medium was removed the next day, replaced with fresh DMEM plus 10% fetal bovine serum and the incubation continued for 24 hours. The cells were fixed and stained with 5-bromo-4-chloro-3-indolyl-β-D-galactopyranoside (40 hours after first exposure to virus). Individual blue cells were counted directly to determine infectious particles per ml (TCIU). Each titer of these viral stocks was performed in triplicate and at least two different titer determinations were performed for each virus preparation.

Intravenous exposure of BLT mice with infectious virus was conducted via tail vein injection with the indicated tissue culture infectious units (TCIU). Viral load in peripheral blood of infected mice was monitored longitudinally by quantitative real-time PCR using Taqman RNA to-C_T_™ 1-step kit from Applied Biosystems, USA [72,73,80]. The sequences of the forward and reverse primers and the Taqman probe for PCR were: 5′-CATGTTTTCAGCATTATCAGAAGGA-3′, 5′-TGCTTGATGTCCCCCCACT-3′, and 5′-FAM CCACCCCACAAGATTTAAACACCATGCTAA-Q-3′, respectively.

CD4^+^ and CD8^+^ T cell levels were monitored by flow cytometric analysis as previously described [40,57,76]. Immunophenotyping was performed on blood samples collected longitudinally and on mononuclear cells isolated from tissues at harvest. Whole peripheral blood (PB) from humanized mice was analyzed according to the BD Biosciences Lyse/Wash protocol (Cat. No. 349202) as we have previously described [81]. Briefly, following antibody labeling of whole blood, red blood cells were lysed. The remaining cells were washed, fixed and the sample was analyzed by flow cytometry. Tissue mononuclear cell isolations and immunophenotyping analyses were also performed according to published methods [40,57,76]. Flow cytometric gating for CD4 and CD8 cell surface expression was performed as follows: (step 1) forward and side scatter properties were utilized to set a live cell gate; (step2) live cells were then analyzed for expression of the human pan-leukocyte marker CD45; (step 3) human leukocytes were then analyzed for hCD3 and (step 4) these T cells or thymocytes were analyzed for hCD4 and hCD8 expression.

The panel of antibodies for analysis of CD8^+^ T cells double positive for CD38^+^ and HLA-DR^+^ was CD8 FITC (SK1), HLA-DR, PE (TU36) or IgG2bκ PE, CD4 PerCP (SK3), CD3 PE-Cy7 (SK7), CD38 APC (HB7) or IgG1κ APC, and CD45 APC-Cy7 (2D1) (all purchased from BD Biosciences). Gating was performed as follows: (step 1) forward and side scatter properties were utilized to set a live cell gate; (step 2) live cells were then analyzed for expression of the human pan-leukocyte marker CD45; (step 3) human leukocytes were then analyzed for CD3; (step 4) T cells were analyzed for CD4 and/or CD8 expression; (step 5) activation of human CD8^+^ T cells was analyzed for HLA-DR and CD38 expression [42]. Gates defining HLA-DR and CD38 expression were set with isotype-matched flourophore-conjugated antibodies.

### Viral replication *in vitro*

The human T-cell line A3.01 (NIH AIDS Reagent Program) was used to propagate both wild-type and *nef*-mutant HIV-1_LAI_. Cells were infected with virus stocks at a multiplicity of infection (MOI) of 0.05 in complete RPMI (containing 10% fetal bovine serum (Hyclone), 50 IU of penicillin per ml, 50 μg streptomycin per ml, 2 mM L-glutamine, and 1 mM sodium pyruvate) plus 2 μg/ml polybrene at 37°C, 5% CO_2_ for 4 hours. The cells were washed extensively with PBS and cultured at 37°C, 5% CO_2_ in complete RPMI. Cell cultures were passaged twice weekly at which time a sample of the culture supernatant was collected for quantification of viral capsid protein by p24^*gag*^ ELISA (HIV-1 p24 Antigen Capture Assay (Advance Biosciences Library, Inc., #5421).

### *In vitro* analysis of Nef activities

The site directed mutations of *nef* in pLAINef*fs*∆-1, pLAINef*fs*∆-13 and pLAINefP72A/P75A were subcloned into pLXSN, a retroviral vector for transduction of CEM T cells and into pcDNA3.1 for transfection into 293T cells [21]. Assays for CD4 downregulation, MHCI downregulation, and activation of PAK2 were described previously [21]. Enhancement of virion infectivity was determined by single infection assays using HeLa-MAGI indicator cells with virus produced from proviral clones transfected into 293T cells [21,82]. Protein expression was determined by Western Blot analysis with sheep anti-Nef antibody or mouse monoclonal anti-Nef [21,83].

### Sequence analysis of plasma virion RNA

Viral RNA was extracted from 20 μl of plasma from infected mice using the QIAamp Viral RNA Mini kit (Qiagen Sciences, USA). RNA was then reverse transcribed into cDNA, which was then subjected to nested PCR. The outer primers for *nef* amplification are 5′-AGCTTGCTCAATGCCACAGCC-3′ and 5′-GCTGCATATAAGCAGCTGCTTTTTG-3′. The inner primers are 5′-TAGAGCTATTCGCCACATACC-3′ and 5′-GCTTGCTACAAGGGACTTTCCGC-3′. Gel purified PCR products were sequenced and the sequences were aligned to HIV_LAI_ sequences to determine if nucleotide changes had occurred.

### Statistics

Mann–Whitney tests were performed in Prism version 5 (Graph Pad). All data plotted as mean ± S.E.M.

## Competing interests

The authors declare that they have no competing interests.

## Authors’ contributions

RLW, WZ, PWD, JFK and JLF performed experiments. JLF, RLW and JVG designed experiments. RLW, PWD, JLF, and JVG analyzed the data. PWD, JLF and JVG wrote the manuscript. All authors read and approved the final manuscript.

## Supplementary Material

Additional file 1 Insertion of a palindromic frame shift into Nef. Click here for file
