# Supplementary material for: In vivo analysis of highly conserved Nef activities in HIV-1 replication and pathogenesis
Source: Retrovirology. 2013 Oct 30;10:125. doi: 10.1186/1742-4690-10-125 (PMC3907037; doi:10.1186/1742-4690-10-125)
Supplement: Additional file 1 — Insertion of a palindromic frame shift into Nef. [file 1742-4690-10-125-S1.docx]

**Additional File 1. Insertion of a palindromic frame shift into Nef.**

XhoI cuts CTCGAG with four base 5’ overhang.

5’CTCGAG3’ 3’GAGCTC5’

Yields

5’C      TCGAG3’ 3’GAGCT      C5’

After generating blunt ends with the Klenow reaction the sequence is:

5’CTCGA   TCGAG3’ 3’GAGCT   AGCTC5’

Religating the plasmid gives a four base insertion:

5’CTCGATCGAG3’ 3’GAGCTAGCTC5’

This process results in a frame shift within a ten base palindrome (starting with nucleotide C8490, K02013). Restoration of wild type function may occur by a four base “skip” by reverse transcriptase. The fact that the sequence is palindromic may increase the probability of a skip for two reasons. First, intra-strand base pairing of the template strand may facilitate a jump from a given nucleotide to one that is four bases downstream. Second, there are five possible four base deletions that will recreate the wild type sequence. These are TCGA, CGAT, GATC, ATCG and TCGA. Note that four base deletions including either the 5’C or the 3’G will restore the reading frame but will not generate the wild type sequence. All four of the reversions to wild type in LAINef*fs* infected mice had wild type sequence (Figure 2A). Reversion of Nef*fs* sequence to wild type will generate a virus with a strong selective advantage over Nef-defective virus.

The infection of mice with LAINef*fs*Δ-1 represents a very different situation. The single base deletion (Δ-1) downstream of the four base insertion reestablishes the reading frame but does not restore the wild type sequence. A four base deletion within the CTCGATCGAG palindrome will yield a virus that retains a frame-shifted *nef* because of the downstream one base deletion of adenine. Thus, a virus with this frame-shifted *nef* will be at a selective disadvantage to LAINef*fs*Δ-1. Similarly, a one base insertion into the four base run of adenines in LAINef*fs*Δ-1 would also make a frame-shifted *nef*. The same considerations apply to LAINef*fs*Δ-13. For these reasons, we considered it likely that LAINef*fs*Δ-1 and LAINef*fs*Δ-13 would be genetically stable during infection during the course of infection.
